# Identifying multiple sclerosis in women of childbearing age in six European countries: a contribution from the ConcePTION project

**DOI:** 10.1007/s10654-025-01264-3

**Published:** 2025-07-18

**Authors:** Marie Beslay, Yvonne Geissbühler, Anna-Belle Beau, Davide Messina, Justine Benevent, Elisa Ballardini, Laia Barrachina-Bonet, Clara Cavero-Carbonell, Alex Coldea, Laura García-Villodre, Anja Geldhof, Rosa Gini, Kerstin Hellwig, Sue Jordan, Maarit K. Leinonen, Sandra Lopez-Leon, Marco Manfrini, Visa Martikainen, Vera R. Mitter, Amanda J. Neville, Hedvig Nordeng, Aurora Puccini, Sandra Vukusic, Joan K. Morris, Christine Damase-Michel

**Affiliations:** 1 CERPOP-SPHERE Team, Toulouse University Hospital, Inserm UMR 1295, Toulouse University, Toulouse, France; 2 Evidence Generation, Novartis Pharma AG, Basel, Switzerland; 3 ARS Toscana, Florence, Italy; 4 Neonatal Intensive Care Unit, Department of Medical Sciences, Centre for Clinical and Epidemiological Research, Emilia Romagna Registry of Birth Defects, University Hospital of Ferrara, University of Ferrara, Ferrara, Italy; 5 Rare Disease Research Unit, Foundation for Promotion of Health and Biomedical Research of Valencian Region (FISABIO), Valencia, Spain; 6 Faculty of Medicine, Health and Life Sciences, Swansea University, Swansea, Wales; 7 Janssen Biologics B.V, Leiden, Netherlands; 8 Department of Neurology, St. Josef-Hospital, Ruhr-University, Bochum, Germany; 9 Department of Data and Analytics, Finnish Institute for Health and Welfare, Helsinki, Finland; 10 Novartis Pharmaceuticals Corporation, East Hanover, USA; 11 Rutgers Center for Pharmacoepidemiology and Treatment Science, Rutgers University, New Brunswick, NJ USA; 12 Department of Medical Sciences, Centre for Clinical and Epidemiological Research, Ferrara University, Ferrara, Italy; 13 School of Health Professionals, Midwifery Department, Bern University of Applied Sciences, Bern, Switzerland; 14 IMER Registry (Emilia Romagna Registry of Birth Defects), University Hospital of Ferrara, Ferrara, Italy; 15 Pharmacoepidemiology and Drug Safety Research Group, Department of Pharmacy, Faculty of Mathematics and Natural Sciences, University of Oslo, Oslo, Norway; 16 Emilia-Romagna Regional Center of Pharmacovigilance, Bologna, Italy; 17 Hospices Civils de Lyon, Hôpital Pierre Wertheimer, Université Claude Bernard Lyon-I, Research on Healthcare Performance, INSERM U1290, Bron, Lyon France; 18 City St George’s, Population Health Research Institute, University of London, London, UK

**Keywords:** Ddisease identification algorithms, Multiple sclerosis, Prevalence, Administrative healthcare data sources, Women of childbearing age, Pregnant women.

## Abstract

Prevalence of Multiple Sclerosis (MS) has increased over the last decades, primarily among women of childbearing age. Several algorithms for identifying MS have been described in the literature, providing heterogeneous prevalence estimates. We compared five algorithms to identify MS in women of childbearing age and estimated MS prevalence by time period and age-group. The study population included women aged 15 to 49 years-old between 2005 and 2019, from three data sources including all women (from Italy, Norway, and Wales), and three including pregnant women only (from France, Finland, and Spain; data collected around pregnancy). Five algorithms were tested: MS1 to MS3 combined MS diagnoses and MS-medicine prescriptions/dispensations, requiring 1, 2, or 3 occurrences, respectively; MS4 and MS5 used only MS diagnoses, requiring at least 2 occurrences (MS4 allowed just 1 if diagnosis was from inpatient care). In 2015–2019, MS prevalence based on MS1 ranged from 109 to 359 per 100,000 women: 109 in France, 121 in Spain, 195 in Wales, 232 in Finland, 264 in Italy, and 359 in Norway. More restrictive algorithms led to greater disparity, with MS3 ranging from 53 in Spain to 325 in Norway, and MS5 from 21 in France to 345 in Norway. All algorithms showed expected prevalence trends by time and age among women of childbearing age, though lower than in the literature. Overall, MS1 provided prevalence estimates most closely aligned with existing literature. This study offers key insights into choosing algorithms for identifying MS in women of childbearing age and in pregnant women.

**Supplementary Information:**

The online version contains supplementary material available at 10.1007/s10654-025-01264-3.

## Background

Multiple sclerosis (MS) is a long-term autoimmune condition affecting around one in 1,000 people worldwide. The prevalence of MS varies within and between countries, being higher in Nordic countries, and has generally increased over the last few decades [1, 2]. Women are two to four times more likely to be affected than men, and are usually diagnosed during their childbearing years, raising the question of the impact of MS and MS treatment on pregnancy [3–5]. Pregnant women are usually excluded from clinical trials, resulting in a lack of information on the safety of use of medication during pregnancy. To fill this gap, post-authorisation observational studies play an essential role. In particular, multicenter studies using data from several healthcare data sources are needed, especially for rare diseases such as MS where data is scarce.

As we show in a concurrent work, the choice of the method for assessing prevalence and the length of the lookback both have an impact on MS prevalence estimates (article in print: DOI 10.1007/s10654-025-01243-8). When estimating MS prevalence in a multicentre study with several healthcare data sources, an additional key factor is the algorithm used to identify MS. Algorithms are useful as they may include diagnoses from different sources, such as inpatient, outpatient, primary care, as well as prescription data [6–10].

A wide range of algorithms for identifying MS in administrative healthcare databases has been described in the literature [6–15]. Capkun et al. tested ten algorithms from the literature in a large US administrative claims database, and the corresponding prevalence estimates ranged from 87 to 212 per 100,000, illustrating the major impact of the choice of the algorithm on prevalence estimate. Based on a comparison with published prevalence, two algorithms appeared to be superior to the others: the first one required two MS diagnoses at least 30 days apart and the second one required at least one principal inpatient MS diagnosis or 2 MS diagnoses at least 30 days apart [7]. However, the choice of the most accurate algorithm can differ depending on the database. In three databases, the preferred algorithm required 3 or more MS-related claims from any combination of inpatient, outpatient, or DMT (Disease-Modifying Therapy) use within 1 year [10]. In Wales, an algorithm requiring either an MS diagnosis code with the disease onset at least six months after the earliest entry in the Welsh Primary Care source, or three MS diagnosis codes, had a sensitivity of 96.8% and a specificity of 99.9% [9]. A less restrictive algorithm, requiring at least one MS record in administrative datasets among medicine prescriptions, hospital discharge and outpatient consultations, was used and validated in several Italian studies, with a sensitivity ranging from 85 to 99% and a specificity ranging from 87.4 to 100% [12–15]. In France, a comparable algorithm requiring only one event among long-term disease status for MS, MS-related hospital admission or reimbursement for MS-specific DMTs was used in the national health data system [8]. The performance of this algorithm was later evaluated, showing a sensitivity of 87.6% and a specificity of 99.9% [11].

Within the ConcePTION project, we aim to explore the use and safety of MS medications during pregnancy using several European healthcare data sources, and the first step is to identify women with MS in these sources. In this study, we aimed to compare 5 algorithms to identify MS among women of childbearing age in six European healthcare data sources. For this purpose, we assessed MS prevalence using these five algorithms, and compared the prevalence estimates within and across data sources. MS prevalence estimates were then compared with published prevalence. Identifying women with MS is a first step to further study the use of MS medicines in women of childbearing age and pregnant women, and the safety of use of these medicines during pregnancy.

Methods.

### Study population

The study population consisted of women aged between 15 and 49 years (i.e. all women of childbearing age including pregnant women), between 2005 and 2019 from six European data sources.

#### Data sources

The study was conducted using health care data sources from six European countries: Finland, Haute-Garonne (France), Emilia Romagna (Italy), Norway, Valencian Region (Spain) and Wales (UK). Detailed information on the data sources are given in online supplementary Table 1. Briefly, in Finland and Norway, data are from administrative healthcare databases with national coverage including birth, prescription, primary and specialized health care registries. The records from all registries are linkable at the individual level by a unique national person identifier. In Haute-Garonne (France), data are from the population-based EFEMERIS cohort of pregnant women living in Haute-Garonne containing data on pregnancy characteristics, outcomes and child health. In Emilia Romagna (Italy) and Valencian Region (Spain), data originate from regional administrative health registries. They include diagnoses from hospital and specialist care contacts (only for the Italian data source) and drug dispensing data. In Wales (UK), data are linked in the SAIL databank [16, 17]; for this study, hospital admissions data (national coverage) was linked with primary care data, including all prescriptions issued in primary care. Some 85% of Wales’ primary care practices contribute data to SAIL.

The Italian, Norwegian, and Wales data sources provided data on women of childbearing age, with complete data coverage during the study period. The Spanish, Finnish, and French data sources provided data only on pregnant women. In Finland, diagnosis data from patient registries was available continuously during the study period, but prescription data was only available from three months prior to pregnancy until three months after the end of pregnancy. In Valencian Region, diagnosis and prescription data was available continuously from 2013 to 2019. In France, the prescription data was available from 2.5 months prior to the pregnancy until the end of pregnancy and maternal diagnostic data (from inpatient data) was available only during the pregnancy.

#### Study period

The study period ran from 1st January 2005 to 31st December 2019. Not all the years were available across all data sources: the exact study periods for each data sources are listed in online supplementary Table 2. Wales data source included historical data from 1 January 1998 to 31 December 2004 for women resident in Wales in the study period.

For each data source including women of childbearing age, the cohort entry date was the latest of the four following dates: the date they joined the data source, the date of their 15th birthday, 1st of Jan of the earliest year of data available in the data source or January 1st, 2005. The cohort exit date was the earliest of the four following dates: the date they left the data source, the date of death, the date of their 50th birthday or December 31st, 2019.

For the data sources including only pregnant women, we restricted data collection to 3 months before to 3 months after pregnancy to be homogeneous between these data source: for Valencian Region (Spain) and Finland, the cohort entry date was 3 months before the 1st day of Last Menstrual Period (LMP) of the first pregnancy and the cohort exit date was 3 months after the end of the last pregnancy; in the French data source, the cohort entry date was 2.5 months before LMP of the first pregnancy and the cohort exit date was the end of the last pregnancy. In these data sources, follow-up could contain several observation periods corresponding to the different pregnancies, separated by periods with no data available. We calculated the coverage of the follow-up, corresponding to the percentage of the follow-up during which the woman is observed.

#### Inclusion criteria

For the data sources including all women of childbearing age (i.e. Italian, Norwegian, and Wales data sources), only women who had complete coverage for at least 365 consecutive days in the study period were eligible.

For the data sources only including pregnant women (i.e. Spanish, Finnish, and French data sources), all complete pregnancy periods lying within the study period for women aged between 15 and 49 years-old during the entire pregnancy period were included in the study. In the Spanish data source, the ConcePTION pregnancy algorithm was used to identify pregnancy episodes, establish the pregnancy type of end and to estimate the pregnancy start date (corresponding to the LMP date) and pregnancy end date [18].

### MS identification algorithms

#### Components of the MS algorithms

Two types of components were used in the algorithms to identify MS: diagnostic codes recorded in various settings, and medicines prescribed or dispensed. Diagnostic codes (listed in online supplementary Table 3) were classified according to their type: inpatient diagnoses (from patients admitted to hospital), primary care diagnoses, and other diagnoses (including diagnoses made during emergency visit or outpatient care). The second component was medications data: dispensing (or prescription in Wales) of MS DMTs (listed in online supplementary Table 4), distinguishing MS-specific DMTs (the only indication is MS) from non-specific MS DMTs (indications for MS and other diseases). The availability of these data components in the six data sources is shown in Table 1.

**Table 1:** Availability of algorithms components in data sources

Country Region	Health care setting	Source for medication data	Presence of data components			
			In-patient diagnoses	Out-patient/ other hospital unspecified diagnoses	Primary care diagnoses	Medication data
Finland *National*	Primary care, out- and in-patient specialist care	Dispensed medicines in community pharmacies	Yes	Yes	Yes	Dispensed
France *Haute-Garonne*	In-patient specialist care	Prescribed and dispensed medicines in community pharmacies	Yes	No	No	Dispensed
Italy *Emilia Romagna*	In-patient specialist care	Dispensed medicines in community and hospital pharmacies (for outpatient use)	Yes	Only from emergency room and mental health service	No	Dispensed
Norway *National*	Primary care, out- and in-patient specialist care	Dispensed medicines in community pharmacies	Yes	Yes	Yes	Dispensed
Spain *Valencian Region*	In-patient specialist care	Dispensed medicines in community and hospital pharmacies (for outpatient use)	Yes	No	No	Dispensed
UK *Wales*	Primary care and in-patient specialist care	Prescribed medicines as recorded in primary care	Yes	No	Yes	Prescribed

#### Algorithm description

Five algorithms to identify MS (named MS1 to MS5) were developed and the estimated prevalences were compared. The algorithm MS1 identified MS cases based on the presence of at least one MS-related diagnosis (all types of care) or at least one prescription for MS-specific DMT, as proposed by Foulon et al. [8]. The algorithm MS2 required to be positive for MS1 and to have one more MS diagnosis or DMT prescription. Based on the study of Culpepper et al. [10], the algorithm MS3 required to be positive for MS2 and to have one more MS diagnosis or DMT prescription. The algorithm MS4 identified MS based on the presence of at least one inpatient MS-diagnosis or at least two outpatient, unspecified or primary-care MS-diagnoses, as proposed by Capkun et al. 2015 [7]. The algorithm MS5 identified MS based on the presence of at least two MS-related diagnosis (all types of care), as proposed by Capkun et al. 2015 [7]. When multiple diagnoses were required, a minimum of 30 days’ separation was required. Table 2 summarizes the criteria required for each algorithm.

**Table 2:** Number and type of events required in the algorithms used to identify women with multiple sclerosis

Algorithm		MS1^4^	MS2		MS3^5^		MS4^6^		MS5^6^
Linkage			AND^3^		AND^3^		OR^3^		
Number of events required Type of event possible		≥1	≥1	≥1	≥1	≥2	≥1	≥2	≥2
MS Diagnoses^1^	Inpatient	✔	✔	✔	✔	✔	✔		✔
	Outpatient/ Hospital unspecified	✔	✔	✔	✔	✔		✔	✔
	Primary care	✔	✔	✔	✔	✔		✔	✔
DMT^2^ prescriptions	MS-specific DMT	✔	✔	✔	✔	✔			
	Non-specific MS DMT			✔		✔			

The table reads as follows: For the algorithm MS2, at least 2 events are required: at least 1 event among MS diagnoses and MS-specific DMTs AND at least one event among MS diagnoses, MS-specific DMTs and non-specific MS DMTs

^1^Diagnosis codes as defined in Table 2

^2^DMT as defined in Table 2

^3^Diagnoses occurring at least 30 days apart

^4^Used by Foulon et al., 2017

^5^Based on Culpepper et al. 2019

^6^Most accurate algorithms according to Capkun et al. 2015

### Statistical analysis

#### Prevalence of MS

Our other study demonstrated that the choice of method for estimating the prevalence of MS can vary significantly depending on the study population (article in print: DOI 10.1007/s10654-025-01243-8). Consequently, we chose to use two different methods to assess prevalence, depending on if the data source included women of childbearing age or pregnant women.

The date of MS identification was the date when the algorithm criteria were met. For example, with the algorithm MS5, 2 MS diagnoses are needed, the date of identification was therefore the date of the second diagnosis. After MS identification, a woman was considered with MS until the end of her follow-up.

In the data sources with women of childbearing age, an average point prevalence of MS was calculated: a point prevalence was calculated on the 1st day of each month during the given period, and the prevalence on the given period was the average of all these points prevalence. On the first day of each month, the MS prevalence was calculated as follow: number of women in the study and identified with MS before or on the given day divided by the number of women in the study on the given day.

In the data sources confined to pregnant women, data were available only during the pregnancy period, making it difficult to identify the exact date of MS diagnosis. Therefore, the date on which the algorithm criteria were met was very unlikely to be the date of first diagnosis. To overcome this lack of precision, we chose to calculate a period prevalence of MS, a method that did not take time into account. Period prevalence of MS over a given period was calculated as follows: the numerator included all women in the study any time during the given period, having been identified with MS before the end of the given period, and the denominator included all women in the study any time during the given period. In contrast to the average point prevalence, when calculating period prevalence over a given period, identification of MS at the end of the given period will have the same weight as an identification of MS before the given period.

Period prevalence over the entire study period, as well as the percentage of variation between prevalence estimates provided by MS2 to MS5 in comparison to prevalence estimates provided by MS1 were also calculated for all the data sources and available in supplementary Table 5.

95% Confidence interval (95% CI) were calculated using the Wilson score method.

#### Covariates

**Fig. 1:**
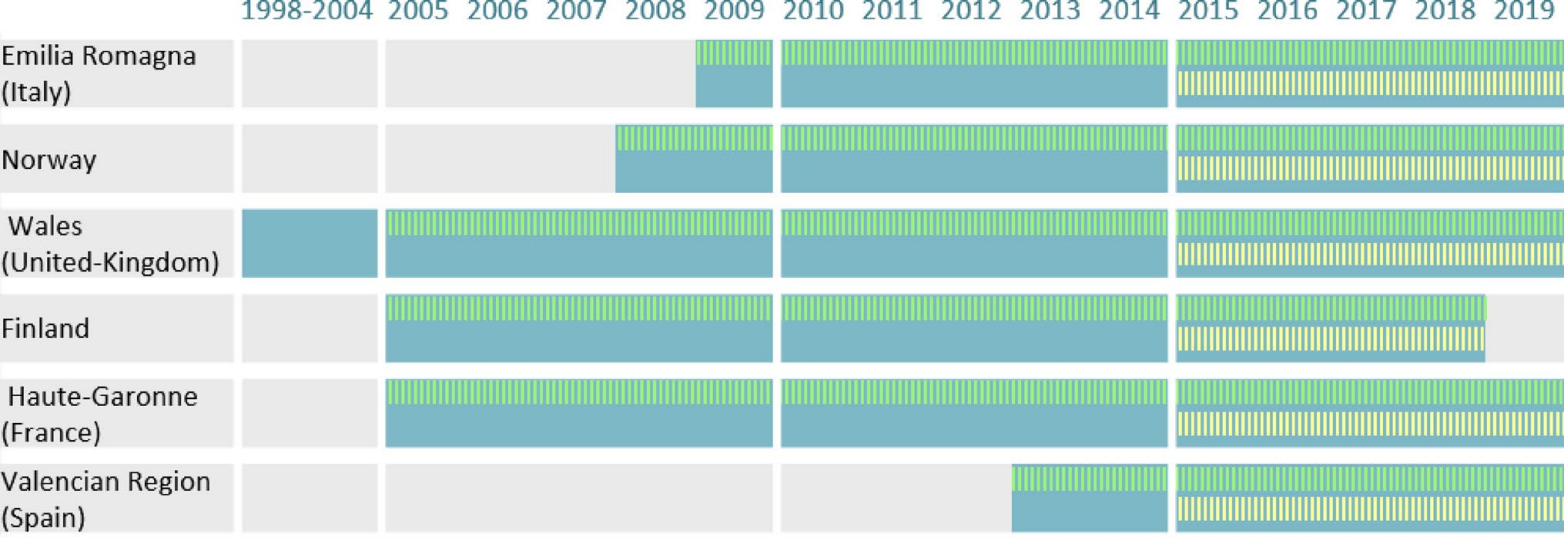
For each data source, periods of available data for identifying Multiple Sclerosis (blue), periods when prevalence is stratified by time period (green stripes), and by age groups (yellow stripes)

Figure 1 illustrates the periods with available data used to identify MS, along with the periods when prevalence stratified by different time intervals and age groups was calculated, by data source.

Prevalence was stratified by five-year intervals (2005–2009,2010–2014,2015–2019). Most data sources covered shorter study periods, resulting in some intervals being less than five years.

Prevalence was also stratified by the age of the woman (15–24,25–29,30–34,35–39,40–49). Results within the 2015–2019 period have been plotted, as this is the most recent period, with the longest lookback available to identify MS.

For the period prevalence, for each age group, women who were into the relevant age group at any time during the period were included in the prevalence calculation. For the average point prevalence, for each age group, women who were into the relevant age group the day of the point prevalence calculation were included in the prevalence calculation.

### Software and common data model

All Data Access Providers (DAPs) extracted an instance from their data source that was large enough to support the study design, and mapped them into the ConcePTION Common Data Model (CDM), thus obtaining an instance of the ConcePTION CDM [19]. This enabled the use of standardized analytics and tools across the network. However, the queries to be executed in distributed analyses still needed to be adapted to the diversity of the data source, including whether the data source could include all women or only pregnant women, the specific coding system, and the specific settings where diagnoses are recorded.

The script was developed using R. A script in SAS was developed to cross-check the outputs of the script within the French data source (EFEMERIS).

The DAPs executed the study code locally on their CDM instance. The result of the script was interpreted and if any inconsistencies were found the script was revised. After reviewing the aggregated results, DAPs approved their upload to the remote Research Environment hosted by the anDREa Consortium, that includes the ConcePTION partner University Medical Center Utrecht. This environment, compliant with local General Data Protection Regulation implementations, could be accessed by the principal investigator.

The results from each of the contributing data sources were then combined in tables and figures for this paper. Non-empty cell counts < 5 were shared in masked format.

## Results

### Description of the population according to the data source

The number of women in the study population in the six data sources, their median time in study, the mean coverage and the number of women diagnosed with MS according to the different algorithms are reported in Table 3. The flowcharts are available in the online supplementary Fig. 1. More than 3,742,000 women of childbearing age were included in the study population, with a median follow-up ranging from 9.1 years in Emilia Romagna (Italy) to 19.7 years in Wales. More than 774,000 pregnant women were included, with a median follow-up ranging from 1 year in France to 2.5 years in Finland. The mean coverage, corresponding to the percentage of the follow-up during which the woman is observed, ranged from 79% in Finland to 96% in Valencian Region (Spain). The maximum relative difference between the number of MS cases captured by the algorithms ranged from 10.4% in Norway to 81.9% in Haute-Garonne (France).

**Table 3:** Description of the study population according to the data source

	Data sources with women of childbearing age			Data sources with pregnant women		
	Emilia Romagna (Italy)	Norway	Wales (United-Kingdom)	Finland	Haute-Garonne (France)	Valencian Region (Spain)
Study population	1,371,568	1,612,782	729,751	482,968	103,330	189,380
Median follow-up (years)	9.1 (4.5-11)	10.5 (5.5-12)	19.7 (14.5-22)^1^	2.5 (1.3-4.8)	1 (1-3)	1.3 (1.2-1.3)
Mean coverage	100%	100%	100%	79%	84%	96%
Number of women meeting the criteria of the 5 MS identification algorithms (N)						
MS1	3,985 (0.29%)	7,351 (0.46%)	1,833 (0.25%)	1,140 (0.24%)	105 (0.10%)	220 (0.12%)
MS2	3,315 (0.24%)	7,106 (0.44%)	1,376 (0.19%)	1,012 (0.21%)	75 (0.07%)	123 (0.06%)
MS3	3,127 (0.23%)	6,586 (0.41%)	1,072 (0.15%)	893 (0.18%)	57 (0.06%)	93 (0.05%)
MS4	2,789 (0.2%)	7,193 (0.45%)	1,473 (0.20%)	954 (0.20%)	67 (0.06%)	200 (0.11%)
MS5	1,281 (0.09%)	7,057 (0.44%)	1,340 (0.18%)	951 (0.20%)	19 (0.02%)	45 (0.02%)
Maximum relative difference between two algorithms	67.8%	10.4%	41.5%	21.6%	81.9%	79.5%

^1^The length of follow-up for Wales considers the historical data available before the study period from 1998

### Prevalence by period

**Fig. 2:**
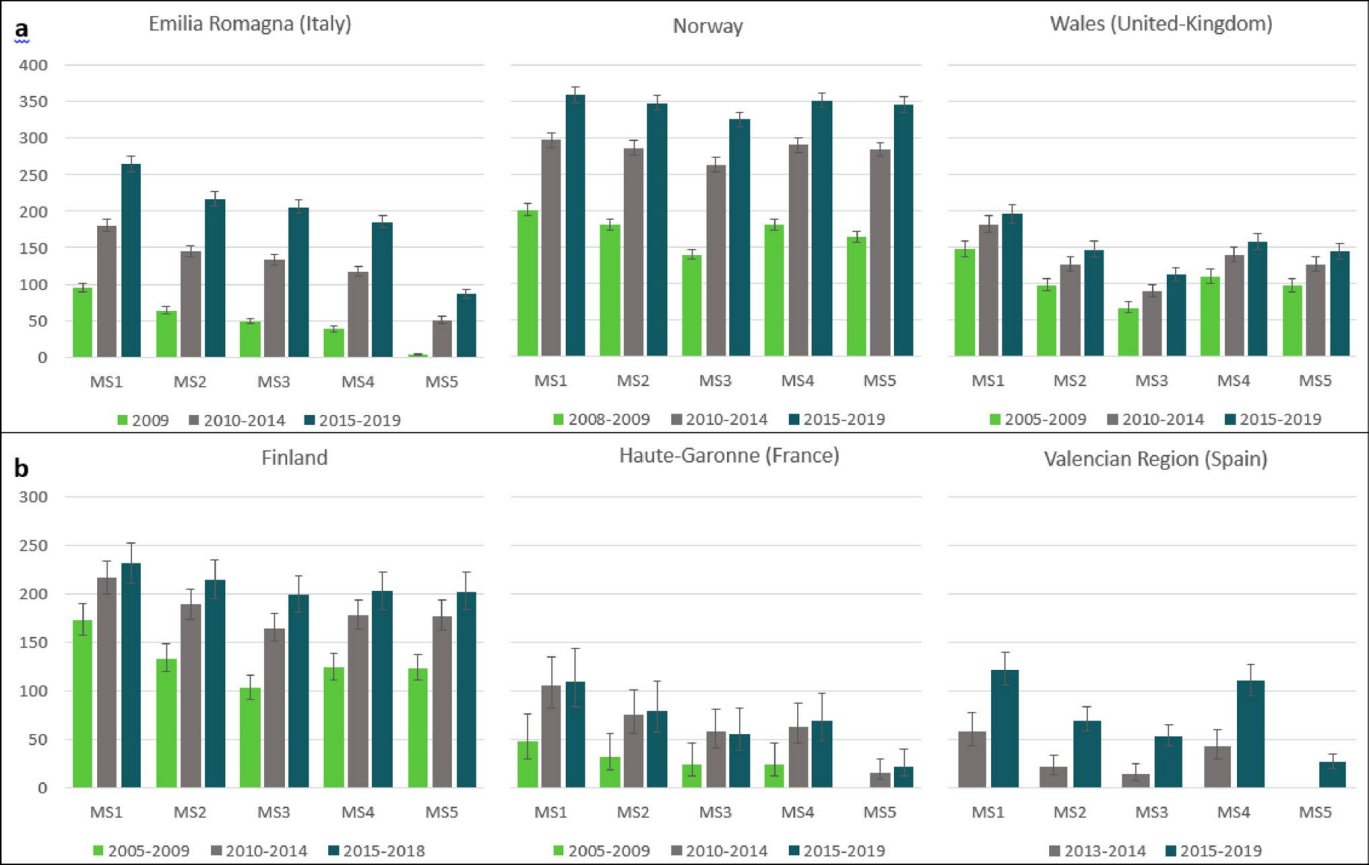
Prevalence of Multiple Sclerosis (MS) per 100,000 women (95% Confidence Interval) according to five MS-identification algorithms (MS1 to MS5), stratified by period in data sources with women of childbearing age (a) and in data sources with pregnant women (b). Prevalence estimates are presented only when at least 5 cases were observed. Values are available in online supplementary Tables 6 and 7. Algorithms MS1 to MS5 are described in Table 2

Prevalence of MS by period among women of childbearing age and pregnant women, according to the five algorithms is shown in Fig. 2. In all the data sources, MS prevalence showed an increase from the period 2005–2009 to the period 2015–2019, with all algorithms. MS1, the least restrictive algorithm requiring one MS diagnosis or one MS-specific medicine prescription or dispensing, provided the highest prevalence in all data sources.

#### In data sources with women of childbearing age

The highest MS prevalence among women of childbearing age was observed in Norway, with prevalence estimates ranging from 201 (95% CI: 193–209) per 100,000 in 2008–2009 to 359 (95% CI: 349–370) per 100,000 women of childbearing age in 2015–2019, with the algorithm MS1. The lowest values in this data source were obtained with the algorithm MS3, with prevalence estimates ranging from 140 (95% CI: 133–147) per 100,000 in 2008–2009 to 325 (95% CI: 315–335) per 100,000 in 2015–2019.

In Emilia Romagna, MS prevalence ranged from 95 (95% CI: 89–101) per 100,000 in 2009 to 264 (95% CI: 254–275) per 100,000 women of childbearing age in 2015–2019, with the algorithm MS1. The lowest values in this data source were obtained with the algorithm MS5, with prevalence estimates ranging from 3 (95% CI: 2–4) per 100,000 in 2009 to 86 (95% CI: 80–92) per 100,000 in 2015–2019.

In Wales, MS prevalence ranged from 147 (95% CI: 136–158) per 100,000 women of childbearing age in 2005–2009 to 195 (95% CI: 183–208) in 2015–2019, with the algorithm MS1. The lowest values were obtained with the algorithm MS3, with prevalence estimates ranging from 66 (95% CI: 59–74) per 100,000 in 2005–2009 to 112 (95% CI: 103–122) per 100,000 in 2015–2019.

#### In data sources with pregnant women

The highest MS prevalence among pregnant women was observed in Finland, with prevalence estimates ranging from 173 (95% CI: 158–190) in 2005–2009 to 232 (95% CI: 212–253) per 100,000 pregnant women in 2015–2018, with the algorithm MS1. The lowest MS prevalence was obtained with the algorithm MS3, with prevalence estimates ranging from 103 (95% CI: 91–116) per 100,000 in 2005–2009 to 199 (95% CI: 181–219) per 100,000 in 2015–2018.

In Haute-Garonne, MS prevalence ranged from 48 (95% CI: 30–76) per 100,000 in 2005–2009 to 109 (95% CI: 83–144) per 100,000 pregnant women in 2015–2019, with the algorithm MS1. The lowest MS prevalence was obtained with the algorithm MS5, with prevalence estimates ranging from 16 (95% CI: 8–30) per 100,000 in 2010–2014 to 21 (95% CI: 12–40) per 100,000 in 2015–2019.

In Valencian Region, MS prevalence ranged from 58 (95% CI: 43–77) per 100,000 pregnant women in 2013–2014 to 121 (95% CI: 106–139) per 100,000 in 2015–2019, with the algorithm MS1. The lowest MS prevalence was obtained with the algorithm MS5, with a prevalence of 26 (95% CI: 20–35) per 100,000 in 2015–2019.

### Prevalence by age group in the period 2015–2019

**Fig. 3:**
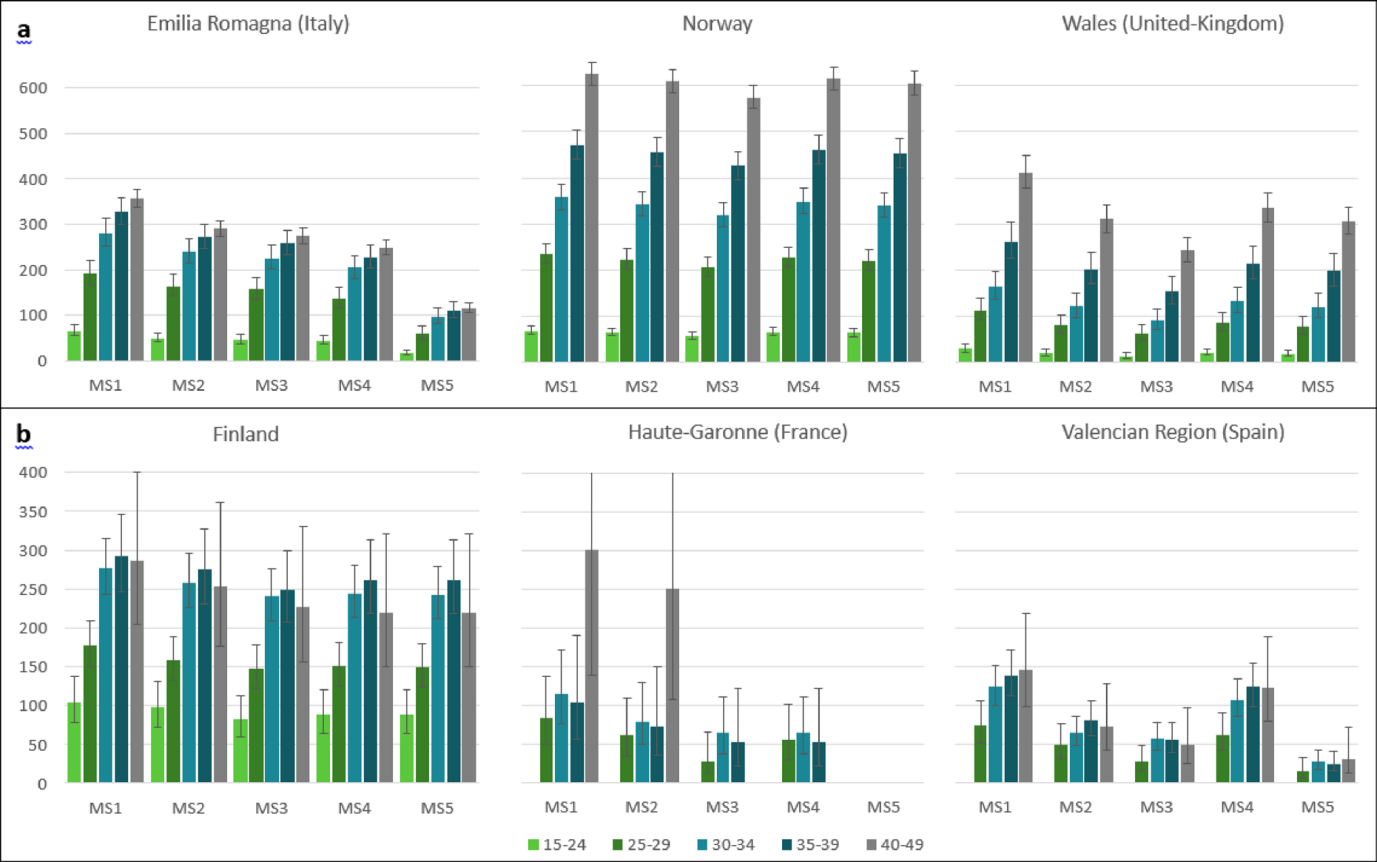
Prevalence of Multiple Sclerosis (MS) per 100,000 women (95% Confidence Interval) according to five MS-identification algorithms (MS1 to MS5), in the 2015-2019 period stratified by age group in data sources with women of childbearing age (a) and in data sources with pregnant women (b). Prevalence estimates are presented only when at least 5 cases were observed. Values are available in online supplementary Tables 8 and 9. Algorithms MS1 to MS5 are described in Table 2

Prevalence of MS by age group according to the algorithms, among women of childbearing age and among pregnant women, is shown in Fig. 3.

#### In data sources with women of childbearing age

In the three data sources with women of childbearing age, MS prevalence increased with age, regardless of algorithm used.

In Emilia Romagna, MS prevalence ranged from 66 (95% CI: 56–79) per 100,000 in women aged 15–24 to 357 (95% CI: 338–377) per 100,000 in women aged 40–49, with the algorithm MS1. MS2 gave the second highest values, with estimates ranging from 50 (95% CI: 41–62) per 100,000 in women aged 15–24 to 290 (95% CI: 273–308) per 100,000 in women aged 40–49. The lowest values were obtained with the algorithm MS5, with prevalence estimates ranging from 17(95% CI: 12–24) per 100,000 in women aged 15–24 to 116 (95% CI: 106–128) per 100,000 in women aged 40–49.

In Norway, MS prevalence ranged from 68 (95% CI: 60–78) per 100,000 in women aged 15–24 to 625 (95% CI: 600–651) per 100,000 in women aged 40–49, with the algorithm MS1. MS4 gave the second highest values, with estimates ranging from 66 (95% CI: 57–75) per 100,000 in women aged 15–24 to 614 (95% CI: 589–640) per 100,000 in women aged 40–49. The lowest values were obtained with the algorithm MS3, with prevalence estimates ranging from 57 (95% CI: 50–66) per 100,000 in women aged 15–24 to 574 (95% CI: 550–599) per 100,000 in women aged 40–49.

In Wales, MS prevalence ranged from 30 (95% CI: 22–40) per 100,000 in women aged 15–24 to 411 (95% CI: 378–447) per 100,000 in women aged 40–49, with the algorithm MS1. MS4 gave the second highest values, with estimates ranging from 21 (95% CI: 15–30) per 100,000 in women aged 15–24 to 334 (95% CI: 305–367) per 100,000 in women aged 40–49. The lowest values were obtained with the algorithm MS3, with prevalence estimates ranging from 13 (95% CI: 8–20) per 100,000 in women aged 15–24 to 242 (95% CI: 217–267) per 100,000 in women aged 40–49.

#### In data sources with pregnant women

In Finland, pregnant women aged between 35 and 39 years-old had the highest prevalence of MS. MS prevalence ranged from 104 (95% CI: 78–138) per 100,000 in women aged 15–24 to 292 (95% CI: 247–346) per 100,000 in women aged 35–39, with the algorithm MS1. The lowest values were obtained with the algorithm MS3, with prevalence estimates ranging from 82 (95% CI: 59–113) per 100,000 in women aged 15–24 to 249 (95% CI: 207–299) per 100,000 in women aged 35–39.

In Haute-Garonne (France), no cases were observed among pregnant women aged between 15 and 24 years-old. MS prevalence increased with age, from the 25–29 to the 40–49 years-old age group. Prevalence ranged from 83 (95% CI: 50–136) per 100,000 in women aged 25–29 to 301 (95% CI: 138–655) per 100,000 in women aged 40–49, with the algorithm MS1. Only 10 women were identified with MS in 2015–2019 with the algorithm MS5, prevalence calculation by age group on age was therefore not possible with this algorithm.

In Valencian Region (Spain), no cases were observed among pregnant women aged between 15 and 24 years-old. MS prevalence increased with age, from the 25–29 to the 40–49 years-old age group. Prevalence ranged from 74 (95% CI: 52–106) per 100,000 in women aged 25–29 to 147 (95% CI: 99–218) per 100,000 in women aged 40–49, with the algorithm MS1. The lowest values were obtained with the algorithm MS5, with prevalence estimates ranging from 15 (95% CI: 7–32) per 100,000 in women aged 25–29 to 31 (95% CI: 13–72) per 100,000 in women aged 40–49.

## Discussion

### Main findings

This study compared five algorithms to identify MS, in three healthcare data sources including women of childbearing age as well as in three healthcare data sources including pregnant women only. As expected, the least restrictive algorithm, MS1, provided the highest prevalence values. By contrast, the algorithm providing the lowest prevalence values was either MS3 or MS5 depending on the data source. Compared to MS1, MS3 required two more events among MS diagnoses and MS-DMT dispensing/prescription. This algorithm returned the lowest prevalence estimates in Norway, Wales and Finland. MS5 required at least two diagnoses for MS, and returned the lowest prevalence values for Emilia Romagna, Haute-Garonne and Valencian Region. Besides variations in prevalence depending on the algorithm used within each data source, differences in MS prevalence were observed between the different data sources. The highest MS prevalence in women of childbearing age and in pregnant women were respectively observed in Norway and Finland, in line with the literature. Conversely, Haute-Garonne (France) and Valencian Region (Spain) had the lowest prevalence values, possibly due to relatively short median follow-ups and more limited data compared to other data sources. These results should be interpreted with caution since a direct validation of the algorithms was not possible, and we therefore cannot rule out false positives and false negatives.

### Comparison of prevalence estimates across data sources

#### MS prevalence increased with period and age in almost all data sources

In all the data sources, an increase of MS prevalence was observed from the 2005–2009 period to the 2015–2019 period, in line with the global rise in MS prevalence reported in the literature [1]. However, as demonstrated in our other study, the prevalence in the first years of the study was underestimated due to the lack of lookback, contributing to the observed increase in prevalence over time (article in print: DOI 10.1007/s10654-025-01243-8).

In addition, in data sources with women of childbearing age, as expected, a clear increase of MS prevalence with age was observed in the three data sources, with all the algorithms. The highest prevalence was therefore observed in the 40–49 years-old age group, in line with the literature [1, 20, 21]. This trend was less evident in data sources limited to pregnant women, especially in French and Spanish data source, probably partly due to the lower number of cases and the resulting low statistical power. In the Finnish dataset, an increase of MS prevalence was observed until 35–39 years-old, which showed the highest prevalence with all algorithms. The lower prevalence in the oldest age group compared to the 35–39 age group might be due to the lower number of women in the 40–49 age group, and the resulting lower statistical power.

#### Shorter follow-up and a fewer identifying variables increased the variability between algorithms

The Norwegian data source provided a wide range of data to identify women with MS, including diagnoses from inpatient, outpatient and primary care, as well as medication data. As a result, the maximum relative difference between two algorithms was 10.4%: 90% of the cases identified by the least restrictive algorithm MS1, were also identified by the most restrictive algorithm in this data source, MS3. In other words, 90% of the women having one MS diagnosis or one dispensing for an MS-specific medicine had at least 2 other events among MS diagnoses and MS-DMT dispensing. By contrast, in Wales, which did not provide outpatient diagnoses, the maximum relative difference between two algorithms was 41.5%: 58.5% of the cases identified by the least restrictive algorithm MS1, were also identified by the most restrictive algorithm, MS3. Finally, the Italian data source provided only diagnoses from inpatient care, mental health service and emergency room, as well as medication data, and differences between the algorithms were even larger. Indeed, only 32% of the women positive to MS1 were also identified by the most restrictive algorithm MS5, requiring 2 diagnoses for MS.

Like the Norwegian database, the Finnish data source also provided a wide range of data to identify MS cases. However, in contrast to the Norwegian source, medication data was available only for pregnant women and around the period of pregnancy. Median follow-up in this population was therefore four times shorter than in Norway (2.5 years) and the maximum relative difference between two algorithms was 21.7%. In the Spanish and French data source, having a median follow-up of 1.3 and 1 year respectively, the maximum relative difference between two algorithms was much greater (79.5% and 81.9% respectively). This can be explained by the fact that the short follow-up period limits the number of events that can be captured. Indeed, as shown in our other study, the proportion of cases identified based on a single event (using the MS1 algorithm) with only one year of data ranged from 44 to 83%, depending on the data source (article in print: DOI 10.1007/s10654-025-01243-8). In addition, disease activity has been shown to decrease during pregnancy and some MS treatment are not recommended, also reducing the chances to detect the disease during this period [12, 22].

#### More follow-up and more identifying variables led to a higher MS prevalence

Consistently, data sources with a longer follow-up and/or more variables available to identify MS also had higher prevalence of MS. For example, prevalence in the Norwegian database (359 per 100,000 with MS1 in 2015–2019 for example) was higher than in Emilia Romagna (264 per 100,000) and Wales (195 per 100,000). Similarly, the prevalence of MS in pregnant women was higher in Finland (232 per 100,000) than in French (109 per 100,000) and Spanish (121 per 100,000) data sources. The less comprehensive variable availability for detecting MS and/or the shorter time windows for case identification could therefore have led to false negatives and therefore to an underestimation of MS prevalence in Wales and, to a greater extent, in Spanish and French data sources. However, as MS prevalence has been shown to be higher in the Nordic countries, the higher prevalence observed in Norway and Finland was expected [2].

### Comparison with published prevalence

We compared our prevalence values with literature in the relevant country and region when available, focusing on MS prevalence among women during the study period. We prioritized studies that used reliable methods for disease identification. All the relevant studies, along with the method used to identify the disease, are described in online supplementary Table 10.

In Norway, several studies published heterogenous prevalence estimates of MS from several regions, from 250 per 100,000 women in 2010 in Nordland County to 473 in 2018 in Møre and Romsdal County [23–27]. At a national level, a prevalence of 280 per 100,000 women was reported in 2012 using principally the Norwegian Patient Registry (NPR) which provides data on inpatient and outpatient visits in secondary care since 2008 [28]. This is however likely to be an underestimate of more than 6% as it did not include individuals diagnosed before 2008 without any MS diagnosis registered in a hospital since then, and it required individuals to have two registrations [29]. The actual prevalence might therefore be closer to the value obtained in 2010–2014 with MS4 (290 per 100,000) or MS1 (297 per 100,000).

In the province of Ferrara in Italy, localized within the Emilia Romagna region covered in our study, a prevalence of 261 per 100,000 women was reported in 2016 [30]. The algorithm MS1 provided a close prevalence estimate (264 per 100,000 women) over the period 2015–2019. In Wales, MS prevalences of approximately 243 per 100,000 women in 2010 and 222 per 100,000 people in 2020, were reported [9, 31]. In this last study, prevalence data for women was not reported separately, but as women are affected more than men, we can assume that the prevalence in women was higher than this value. In comparison, in our study, the least restrictive algorithm MS1 provided the closest prevalence estimates (180.6 per 100,000 in 2010–2014 and 195 in 2015–2019) in Wales.

For data sources involving pregnant women only, we also compared our results with published MS prevalence in women, as there are, to our knowledge, no existing MS prevalence data specifically in pregnant women in the relevant countries. This, combined with the short timeframe available to identify the disease in these data sources, probably contributed to the lower prevalence estimates observed in our study compared to the literature. In Finland, based on the data from the Finnish MS register, of a population of 5.5 million in 2018, more than 7,150 women had MS, corresponding to a prevalence of 260 per 100,000 women [32]. In our study, in the Finnish data source, a prevalence of 232 per 100,000 pregnant women in 2015–2018 was obtained with the algorithm MS1. In the French and Spanish data sources, MS prevalence was much lower than the published prevalence, probably reflecting an even higher underestimation of this value due to the absence of outpatient and primary care diagnoses data in these data sources. In France, Foulon et al. reported a prevalence of 195.6 per 100 000 women in 2012 in Haute-Garonne, the area covered in our study [8]. In our study, a prevalence of 105 per 100,000 pregnant women was obtained in Haute-Garonne with the algorithm MS1 in 2010–2014. In the Valencian Region, an MS prevalence of 153 per 100,000 women was reported in 2021, based on diagnoses from primary care [33]. In our study, a prevalence of 121 per 100,000 pregnant women was obtained with the algorithm MS1 in 2015–2019 in the data source covering the same region. In the United States, an MS prevalence of 130 per 100,000 pregnant women was reported in the Truven Health cohort, where a diagnosis code was required on at least 2 unique days from 90 days before LMP to the delivery date [34]. In comparison, in 2010–2014, with the algorithm MS5 also requiring 2 diagnosis codes, we obtained a prevalence of 177.5 per 100,000 in the Finnish data source and 15.8 per 100,000 in the French data source.

### Strengths and limitations

#### Strengths

The main strength of this study is the use of diverse data sources from multiple healthcare systems and populations, enabling a broader picture of the prevalence of MS among women in Europe than any other previous study. A similar methodology was applied across all data sources: all data sources were converted to a same CDM, which was designed to preserve data diversity; the same script was used across data sources, implementing the same algorithms to identify MS cases. Observed heterogeneous results can then be interpreted either as diversity in the data source, or as true differences in the population, and not as different interpretation or implementation of the study protocol. In addition, the algorithms tested in our study are based on previous literature and adapted to the data source used. Indeed, the algorithms used all potential disease-identifying variables in each data source. Another strength is the use of a method to calculate prevalence tailored to the type of data source. Indeed, based on our other study exploring the impact of prevalence calculation methods on MS prevalence estimates within the same data sources, MS prevalence was estimated using two different methods according to the type of data source. It should however be noticed that the average point prevalence method used in the population of women of childbearing age might underestimate prevalence estimates, particularly at the start of the study, due to the delay between diagnosis of the disease and detection by our algorithm. On the other hand, the period prevalence used in the pregnant women population might slightly overestimate prevalence estimates.

#### Limitations

The major limitation of this study is the impossibility of validating the algorithms tested, due to the use of administrative data sources. However, a comparison between estimates of prevalence using the different algorithms within and across data sources, as well as a comparison with published prevalence, allowed to identify algorithms providing the most probable prevalence estimates. There are, however, several limitations when comparing our study to the existing literature. First, the study populations often differed: we focused on women of childbearing age or only pregnant women, aged 15 to 49 years-old, whereas published prevalence estimates are usually among all women, with sometimes stratification by age group. Second, published studies may not always use the best method for identifying MS, which can affect their reliability as references for comparison. Different diagnostic criteria, data source, and healthcare access can impact the reported prevalence. To overcome these limitations, we have given priority to studies from the same area with data from registries and/or to studies where MS cases were validated by experts. Finally, variations in data coverage and lookback period available across different data sources may introduce biases and affect the reliability of our prevalence estimates. Therefore, even if the least restrictive algorithm (MS1), requiring one MS diagnosis or one dispensing/prescribing for MS-specific DMT, was the closest to published prevalence estimates, we cannot rule out false positive diagnoses occurring in administrative data due to tentative diagnoses or typing errors. Indeed, misdiagnosis and off-label use of MS-specific DMTs for causes other than MS may have led to false positives. Also, the lack of outpatient data for the Wales and Italian datasets might have led to false negatives. On the other hand, even with the algorithm MS1, our prevalence estimates were mostly lower than published prevalence, especially in data sources with pregnant women only, probably reflecting an underestimation of MS prevalence due to false negatives.

## Conclusion

This study aimed to compare five algorithms to identify MS among women of childbearing age in six European healthcare data sources. In data sources with women of childbearing age, the five algorithms provided expected prevalence trends regarding variation with time and age. The least restrictive algorithm (MS1), which required only one MS diagnosis or one dispensing for MS-specific DMT, provided prevalence estimates that most closely aligns with existing literature. During the 2015–2019 period, this algorithm provided a prevalence of 359 per 100,000 in Norway, 264 per 100,000 in Emilia Romagna (Italy), and 195 per 100,000 in Wales (UK) among women of childbearing age, and a prevalence of 232 per 100,000 in Finland, 121 per 100,000 in Valencian Region (Spain) and 109 per 100,000 in Haute-Garonne (France) among pregnant women. However, these prevalence estimates should be interpreted with caution since a direct validation of the algorithms was not possible, and we therefore cannot rule out false positives and false negatives. The choice of algorithm must be aligned with the specific objectives of the study: for applications where high confidence in MS diagnosis is essential, such as evaluating healthcare practices or clinical or therapeutic management, more restrictive algorithms are preferable, as they minimize false positives by requiring multiple events; on the other hand, if the objective is to identify the greatest possible number of MS cases, to produce reliable estimates of prevalence for example, less restrictive algorithms are preferable due to their higher sensitivity. This study demonstrates how different algorithms can be used to identify multiple sclerosis in women of childbearing age and pregnant women within healthcare data sources. It also provides new prevalence data for MS in European countries with good geographic spread. These insights could contribute to further research on MS medication use in women of childbearing age and pregnant women and on the safety of use of MS medication during pregnancy.

## Electronic supplementary material

Below is the link to the electronic supplementary material.

Supplementary Material 1

Supplementary Material 2

## Data Availability

All relevant data are within the paper and its Supporting Information files. Authors may not share the study data due to regulations which restrict access and distribution to those with ethical and legal permission to use the data. The study material is available to other researchers upon an application to relevant register holders. The study protocol was registered in the HMA-EMA Catalogue (EUPAS43420) and is available on zenodo repository [35]. All code lists and scripts can be found at (10.5281/zenodo.15355612; https://github.com/IMI-ConcePTION/DP3-MS-SLE).
