# Supplementary material for: Identifying multiple sclerosis in women of childbearing age in six European countries: a contribution from the ConcePTION project
Source: Eur J Epidemiol. 2025 Jul 18;40(8):891–904. doi: 10.1007/s10654-025-01264-3 (PMC12374901; doi:10.1007/s10654-025-01264-3)
Supplement: Supplementary file 2 — Supplementary Material 2 [file 10654_2025_1264_MOESM2_ESM.docx]

**Supplementary information**

Article title: Identifying multiple sclerosis in women of childbearing age in six European countries: A contribution from the ConcePTION project

Authors: Marie Beslay, Yvonne Geissbühler, Anna-Belle Beau, Davide Messina, Justine Benevent, Elisa Ballardini, Laia Barrachina-Bonet, Clara Cavero-Carbonell, Alex Coldea, Laura García-Villodre, Anja Geldhof, Rosa Gini, Kerstin Hellwig, Sue Jordan, Maarit K Leinonen, Sandra Lopez-Leon, Marco Manfrini, Visa Martikainen, Vera R Mitter, Amanda J Neville, Hedvig Nordeng, Aurora Puccini, Sandra Vukusic, Joan K Morris, Christine Damase-Michel

Corresponding author:

Marie Beslay, Toulouse University Hospital, CERPOP-SPHERE Team, Inserm UMR 1295, Toulouse University, Toulouse, France

beslay.m@chu-toulouse.fr

Journal: European Journal of Epidemiology

## ****Supplementary table 1.** Description of participating data sources**

| **Country / Coverage** | **Name or registers** | **Description** |
| --- | --- | --- |
| Finland / *National* | Linkage of national registries: Medical birth registry, Prescription drug registry, National patient registry, Register of Primary Health Care visits | **Medical birth registry:** Information on births, pregnancy length, maternal parity, smoking, maternal age, maternal comorbidity, and pregnancy and birth complications.  **Prescription drug registry**: Data on all prescription entitled to reimbursement fills from pharmacies including Anatomical therapeutical chemical (ATC) classification codes and dispensing date. Over the counter medicines and medicines used during in-patient hospital stays are not included. Data are available from three months before to three months after pregnancy.  **National patient registry**: Health administrative registry from private and public specialist care. Data on inpatient stays and outpatient care contacts included diagnoses coded according to International Statistical Classification of Diseases and Related Health Problems revision 10 (ICD-10).  **Register of Primary Health Care visits:** Health administrative registry from public health care centres and increasingly from private service providers. Data on primary care visits contacts included diagnoses coded according to International Statistical Classification of Diseases and Related Health Problems revision 10 (ICD-10) or International Classification of Primary Care, second edition (ICPC-2). |
| France / *Haute-Garonne region* | EFEMERIS database | EFEMERIS database includes data on pregnant women covered by the French Health Insurance System in Haute-Garonne (south-west France). Specifically, EFEMERIS comprises data about (a) all prescriptions redeemed at pharmacies by women in ambulatory care, prior to and during pregnancy [names, Anatomical, Therapeutic and Chemical classification system (ATC) and dispensing dates]  (b) the mother (age, level of education…) and the child (gender, birth size, birth weight, congenital anomalies…) through children’s certificates filled out during the compulsory medical examinations at birth, 9 and 24 months, (c) Terminations Of Pregnancy for Fetal Anomaly (TOPFAs) that has been considered in the maternities of the region, (d) nature and date of termination of pregnancy (elective termination, stillbirth, and spontaneous abortion) from the Toulouse University Hospital centre, and (e) data on inpatient stays during pregnancy included diagnoses coded according to International Statistical Classification of Diseases and Related Health Problems revision 10 (ICD-10). |
| Italy / *Emilia Romagna region* | Linkage of Emilia-Romagna administrative databases and registries. Inhabitant registry, drug dispensations from community pharmacies and from hospital pharmacies, hospital discharge records, emergency admissions, outpatient services, exemptions from copayment, mental health services, birth registry, and death registry | Emilia-Romagna registries include birth, death, and anagraphic registries. Birth registry collect information on births, type of pregnancy, date of delivery, type of pregnancy outcome, pregnancy length, weight at birth, maternal age. Administrative databases collect information about drug dispensation from community and hospital pharmacy including ATC code and dispensing quantity and date. Hospital discharge record collect data about inpatients stay, including diagnosis (ICD9) as well as emergency admission database and mental health services databases collect data from outpatients. Moreover, exemptions from copayment databases collect information about exemption related to specific diagnostic code. |
| Norway / *National* | Linkage of national registries: Medical birth registry, Prescription drug registry, National patient registry, Register of Primary Health Care visits | **Medical birth registry:** Information on births, term data, pregnancy length, maternal parity, smoking, maternal age, maternal comorbidity, and pregnancy- and birth complications.  **Prescription drug registry (from 2005)**: Data on all prescription fills from pharmacies including Anatomical therapeutical chemical (ATC) classification codes and dispensing date. Over the counter medicines and medicines used during in-patient hospital stays are not included.  **National patient registry (from 2008)**: Health administrative registries from private and public specialist care. Data on inpatient stays in hospitals and outpatient care contacts from specialists included diagnoses coded according to International Statistical Classification of Diseases and Related Health Problems revision 10 (ICD-10).  **Register of Primary Health Care visits (from 2008):** Health administrative registries from primary care. Data on primary care contacts including outpatient specialists (data overlapping with the National patient registry). Diagnostic codes follow the International Classification of Diseases, version 10 (ICD-10) and International Classification of Primary Care (ICPC-2) which is more frequently used by general practitioners. |
| Spain / *Valencian Region* | Linkage of administrative databases and registries:  Perinatal Mortality Registry (RMPCV); Birth Registry (META-B); Congenital Anomaly Population-based Registry (RPAC-CV); Prescription and dispensations dataset (GAIA); Morbidity- Hospital discharges database (CMBD) | **Birth Registry** (for live births), **Perinatal Mortality Registry** (for stillbirths and live births deaths during the first 28 days of life) and **Congenital Anomaly Population-based Registry** (for stillbirths and live births with at least 1 major congenital anomaly): Information on births, type of pregnancy, date of delivery, type of pregnancy outcome, pregnancy length, weight at birth, maternal age.  **Prescription and dispensations** **dataset**: Data on all prescription and dispensation to outpatients entitled to reimbursement fills from pharmacies including Anatomical therapeutic chemical (ATC) classification codes and dispensing date.  **Morbidity- Hospital discharges database**: Health administrative registries from private and public inpatients care. Data on inpatient stays included diagnoses coded according to International Classification of Diseases 10th revision Spanish Version (ICD-10ES). |
| UK / *Sample of Wales population* | SAIL databank, including in-patient and primary care records, emergency care, ONS births and deaths, and data (including all prescriptions and vaccinations) from ~85% of Wales GP practices | National, whole-population data, with the exception of primary care practices. Practices contribute voluntarily, without additional funding. For details see Ford et al 2009, Lyons et al 2009. |

****Supplementary table 2****. Study population and study period in the six data sources

| Country/Region | Study population | Study period |
| --- | --- | --- |
| Italy/Emilia Romagna | Women of childbearing age (15-49) | 01/01/2009 -31/12/2019 |
| Norway/ National |  | 01/01/2008 -31/12/2019 |
| UK/Sample of Wales population (70%) |  | 01/01/2005 -31/12/2019^1^ |
| Finland/ National | Pregnant women aged 15-49 (data from 3 months before LMP to 3 months after end of pregnancy^2^) | 01/01/2005 -31/12/2018 |
| Spain/Valencia Region |  | 01/01/2013 -31/12/2019 |
| France/Haute-Garonne |  | 01/01/2005 -31/12/2019 |

^1^ In Wales, additional historical data were available from 01/01/1998 for women in the study population

^2^ From 2.5 months before pregnancy to end of pregnancy for the French data source

NA = Not available

****Supplementary table 3****. MS diagnosis codes

| Coding system | Code | Text Description |
| --- | --- | --- |
| ICD-9 | 340 | Multiple sclerosis |
| ICD-10/ ICD10ES | G35 | Multiple sclerosis |
| ICPC-2 | N86 | Multiple sclerosis |
| READ | 666A. | Multiple sclerosis review |
|  | 666B. | Multiple sclerosis multidisciplinary review |
|  | 8Cc0. | Management of multiple sclerosis in onset phase |
|  | 8Cc1. | Management of multiple sclerosis in early disease phase |
|  | 8Cc2. | Management of multiple sclerosis in stable disability phase |
|  | 8Cc4. | Management of multiple sclerosis in palliative phase |
|  | 8CS1. | Multiple sclerosis care plan agreed |
|  | 8IAb. | Multiple sclerosis review declined |
|  | 9kG. | Spec serv for pat with multiple sclerosis - enh serv admin |
|  | 9mD0. | Multiple sclerosis monitoring first letter |
|  | 9mD1. | Multiple sclerosis monitoring second letter |
|  | 9mD.. | Multiple sclerosis monitoring administration |
|  | F200. | Multiple sclerosis of the brain stem |
|  | F201. | Multiple sclerosis of the spinal cord |
|  | F202. | Generalised multiple sclerosis |
|  | F203. | Exacerbation of multiple sclerosis |
|  | F204. | Benign multiple sclerosis |
|  | F206. | Primary progressive multiple sclerosis |
|  | F207. | Relapsing and remitting multiple sclerosis |
|  | F208. | Secondary progressive multiple sclerosis |
|  | F20. | Multiple sclerosis |
|  | F20z. | Multiple sclerosis NOS |

****Supplementary table 4****. Disease modifying therapies used to treat MS

| **Medication group** | **Medication** | **Code** |
| --- | --- | --- |
| MS-specific DMT (indicated only for MS) | Alemtuzumab (from 2013 onwards)^1^ | L04AA34/L04AG06 |
|  | Daclizumab (from 2016 onwards)^2^ | L04AC01/L04AA08 |
|  | Dimethyl fumarate (until end of May 2017)^3^ | N07XX09/ L04AX07 |
|  | Fingolimod | L04AA27/L04AE01 |
|  | Glatiramer acetate | L03AX13 |
|  | Interferon beta-1a | L03AB07 |
|  | Interferon beta-1b | L03AB08 |
|  | Ocrelizumab | L04AA36/L04AG08 |
|  | Peginterferon beta-1a | L03AB13 |
|  | Teriflunomide | L04AA31/L04AK02 |
|  | Siponimod | L04AA42/L04AE03 |
| Non-specific MS DMT (with other indications) | Dimethyl fumarate (from June 2017 onwards)^3^ | N07XX09/ L04AX07 |
|  | Azathioprine | L04AX01 |
|  | Cladribine | L04AA40/L01BB04 |
|  | Natalizumab^4^ | L04AA23/L04AG03 |
|  | Mitoxantrone | L01DB07 |
|  | Rituximab | L01XC02/L01FA01 |
|  | Methotrexate | L04AX03/L01BA01 |
|  | Cyclophosphamide | L01AA01 |
|  | Leflunomide | L04AA13/L04AK01 |
|  | Mycophenolate mofetil | L04AA06 |
|  | Tacrolimus | L04AD02/D11AX14/D11AH01 |

*^1^Between 2001 and 2012 Aletuzumab was approved by EMA to treat B-cell chronic lymphocytic leukaemia. The approval of Alemtuzumab in MS treatment by EMA was in 2013*

*^2^Between 1999 and 2009 Daclizumab was approved by EMA in organ rejection in de novo allogenic renal transplantation. The approval of Daclizumab in MS treatment by EMA was in 2016*

*^3^ In June 2017, Dimethyl Fumarate was approved to treat Psoriasis by EMA. Dimethyl Fumarate will be considered specific for MS until the end of September 2017, and non-specific from October 2017 onwards for Spain (national approval in October); for Finland (national approval 6 months before end of study period), France (not authorised) and Italy (national approval 3 months before end of study period), Dimethyl Fumarate will be considered MS-specific during the entire period*

*^4^ Natalizumab is only indicated for MS but can also be used by people with for inflammatory bowel diseases*

**
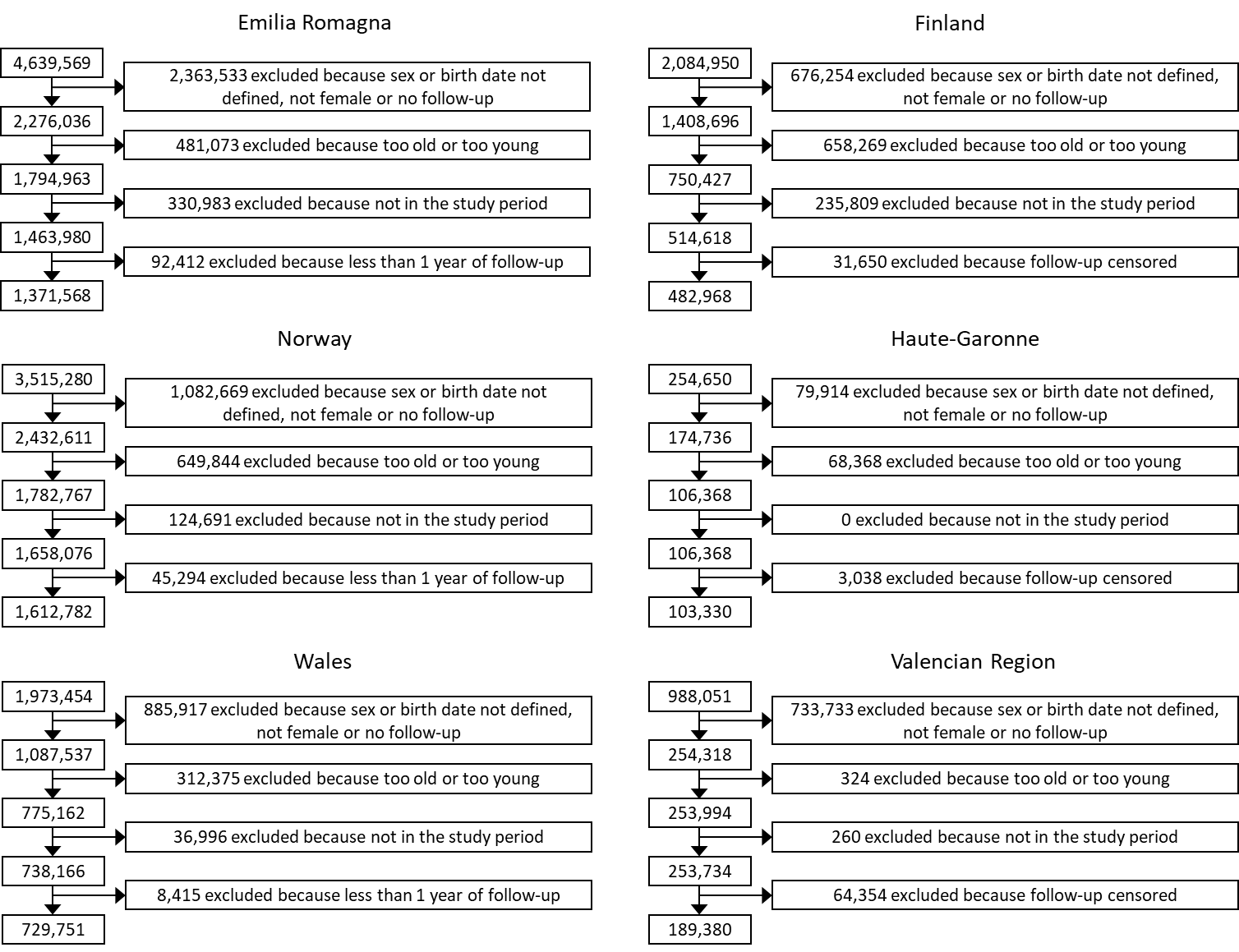
**

**Figure 1.** Flowchart for the selection of women of childbearing age and pregnant women by data source

**Supplementary table 5. MS Period Prevalence over the study period and relative difference between prevalence estimates provided by MS2 to MS5 in comparison to prevalence estimates provided by MS1.**

|  | **Data sources with women of childbearing age** | | | **Data sources with pregnant women** | | |
| --- | --- | --- | --- | --- | --- | --- |
|  | **Emilia Romagna**  **(Italy)** | **Norway** | **Wales (United-Kingdom)** | **Finland** | **Haute-Garonne**  **(France)** | **Valencian Region**  **(Spain)** |
| **Study period** | 2009-2019 | 2008-2019 | 2005-2019 | 2005-2018 | 2005-2019 | 2013-2019 |
| **MS1** | 290.5 (Ref) | 455.8 (Ref) | 251.2 (Ref) | 236 (Ref) | 101.6 (Ref) | 116.2 (Ref) |
| **MS2** | 241.7 (16.8%) | 440.6 (3.3%) | 188.6 (24.9%) | 209.5 (11.2%) | 72.6 (28.6%) | 64.9 (44.2%) |
| **MS3** | 228.0 (21.5%) | 408.4 (10.4%) | 146.9 (41.5%) | 184.9 (21.7%) | 55.2 (45.7%) | 49.1 (57.8%) |
| **MS4** | 203.3 (30.0%) | 446.0 (2.2%) | 201.8 (19.6%) | 197.5 (16.3%) | 64.8 (36.2%) | 105.6 (9.1%) |
| **MS5** | 93.4 (67.8%) | 437.6 (4.0%) | 183.6 (26.9%) | 196.9 (16.5%) | 18.4 (81.9%) | 23.8 (79.5%) |

****Supplementary t****able 6. Number of MS cases and average point prevalence per 100,000 women (95% Confidence Interval) stratified by five-years periods^1^ in data sources with women of childbearing age

|  |  | **MS1** | | **MS2** | | **MS3** | | **MS4** | | **MS5** | |
| --- | --- | --- | --- | --- | --- | --- | --- | --- | --- | --- | --- |
| Period | Population | Cases | Prevalence | Cases | Prevalence | Cases | Prevalence | Cases | Prevalence | Cases | Prevalence |
| ***Italy (Emilia Romagna)*** | | | | | | | | | | | |
| 2009 | 997,136 | 1,237 | 94.6 (89-100.7) | 952 | 63.7 (59.3-68.6) | 864 | 48.9 (45.2-53) | 605 | 38.2 (34.7-42.1) | 93 | 3.2 (2.4-4.4) |
| 2010-2014 | 1,197,622 | 2,638 | 180.3 (172.1-188.8) | 2,128 | 144.6 (137.3-152.2) | 1,977 | 133.2 (126.2-140.5) | 1,868 | 117.1 (110.6-124) | 849 | 50.8 (46.6-55.4) |
| 2015-2019 | 1,138,932 | 3,506 | 264.3 (254.1-274.9) | 2,918 | 217.3 (208.1-226.9) | 2,773 | 205.6 (196.6-215) | 2,454 | 185.3 (176.8-194.2) | 1,116 | 85.9 (80.2-92.1) |
| ***Norway*** | | | | | | | | | | | |
| 2008-2009 | 1,146,745 | 3,039 | 200.7 (192.6-209) | 2,894 | 180.1 (172.5-187.9) | 2,510 | 139.8 (133.3-146.6) | 2,935 | 180.1 (172.6-188) | 2,848 | 163.7 (156.6-171.1) |
| 2010-2014 | 1,363,982 | 5,000 | 296.6 (286.8-306.6) | 4,845 | 285.9 (276.3-295.8) | 4,499 | 262.9 (253.8-272.4) | 4,898 | 289.7 (280.1-299.6) | 4,812 | 283.8 (274.3-293.7) |
| 2015-2019 | 1,417,710 | 6,121 | 358.9 (348.5-369.7) | 5,925 | 347.5 (337.3-358.1) | 5,540 | 324.6 (314.7-334.8) | 5,989 | 351 (340.7-361.7) | 5,884 | 344.9 (334.7-355.4) |
| ***United-Kingdom (Wales)*** | | | | | | | | | | | |
| 2005-2009 | 523,889 | 920 | 146.5 (135.7-158.1) | 626 | 97.5 (88.8-107) | 443 | 66.2 (59.1-74.1) | 697 | 109.5 (100.3-119.6) | 622 | 96.7 (88.1-106.2) |
| 2010-2014 | 583,864 | 1,250 | 180.6 (169.1-192.9) | 911 | 126 (116.4-136.3) | 666 | 88.9 (81-97.7) | 998 | 139.3 (129.2-150.2) | 903 | 125.3 (115.7-135.6) |
| 2015-2019 | 594,842 | 1,393 | 195.4 (183.4-208.1) | 1,062 | 146.2 (135.9-157.3) | 838 | 111.9 (102.9-121.7) | 1,126 | 157.1 (146.4-168.6) | 1,030 | 143.6 (133.4-154.6) |

^1^ Some periods can be less than 5 years, depending on the data availability in each data source, the exact period is indicated in each row

****Supplementary t****able 7. Number of MS cases and period prevalence per 100,000 women (95% Confidence Interval) stratified by five-years periods^1^ in data sources with pregnant women

|  |  | **MS1** | | **MS2** | | **MS3** | | **MS4** | | **MS5** | |
| --- | --- | --- | --- | --- | --- | --- | --- | --- | --- | --- | --- |
| Period | Population | Cases | Prevalence | Cases | Prevalence | Cases | Prevalence | Cases | Prevalence | Cases | Prevalence |
| ***Finland*** | | | | | | | | | | | |
| 2005-2009 | 258,122 | 447 | 173.2 (157.9-190) | 345 | 133.7 (120.3-148.5) | 265 | 102.7 (91-115.8) | 321 | 124.4 (111.5-138.7) | 319 | 123.6 (110.8-137.9) |
| 2010-2014 | 295,289 | 640 | 216.7 (200.6-234.2) | 558 | 189 (173.9-205.3) | 486 | 164.6 (150.6-179.9) | 526 | 178.1 (163.6-194) | 524 | 177.5 (162.9-193.3) |
| 2015-2018 | 208,128 | 482 | 231.6 (211.8-253.2) | 446 | 214.3 (195.3-235.1) | 414 | 198.9 (180.7-219) | 422 | 202.8 (184.3-223) | 421 | 202.3 (183.9-222.5) |
| ***France (Haute-Garonne)*** | | | | | | | | | | | |
| 2005-2009 | 37,667 | 18 | 47.8 (30.2-75.5) | 12 | 31.9 (18.2-55.7) | 9 | 23.9 (12.6-45.4) | 9 | 23.9 (12.6-45.4) | <5 | NC |
| 2010-2014 | 57,140 | 60 | 105 (81.6-135.1) | 43 | 75.3 (55.9-101.3) | 33 | 57.8 (41.1-81.1) | 36 | 63 (45.5-87.2) | 9 | 15.8 (8.3-29.9) |
| 2015-2019 | 46,625 | 51 | 109.4 (83.2-143.8) | 37 | 79.4 (57.6-109.4) | 26 | 55.8 (38.1-81.7) | 32 | 68.6 (48.6-96.9) | 10 | 21.4 (11.7-39.5) |
| ***Spain (Valencian Region)*** | | | | | | | | | | | |
| 2013-2014 | 79,693 | 46 | 57.7 (43.3-77) | 17 | 21.3 (13.3-34.2) | 11 | 13.8 (7.7-24.7) | 34 | 42.7 (30.5-59.6) | <5 | NC |
| 2015-2019 | 167,294 | 203 | 121.3 (105.8-139.2) | 117 | 69.9 (58.4-83.8) | 89 | 53.2 (43.2-65.5) | 184 | 110 (95.2-127.1) | 44 | 26.3 (19.6-35.3) |

^1^ Some periods can be less than 5 years, depending on the data availability in each data source, the exact period is indicated in each row; NC= Non-calculable

****Supplementary t****able 8. Number of MS cases and average point prevalence per 100,000 women in the 2015-2019 period stratified by age group in data sources with women of childbearing age

|  |  | **MS1** | | **MS2** | | **MS3** | | **MS4** | | **MS5** | |
| --- | --- | --- | --- | --- | --- | --- | --- | --- | --- | --- | --- |
| Age group | Population | Cases | Prevalence | Cases | Prevalence | Cases | Prevalence | Cases | Prevalence | Cases | Prevalence |
| ***Italy (Emilia Romagna)*** | | | | | | | | | | | |
| 15-24 | 301,786 | 310 | 66.1 (55.5-78.7) | 246 | 50.3 (41.2-61.5) | 234 | 46.3 (37.6-57.1) | 213 | 44.2 (35.7-54.7) | 74 | 16.9 (12-23.9) |
| 25-29 | 233,889 | 529 | 192.5 (168.3-220.3) | 461 | 164.6 (142.3-190.5) | 442 | 158.3 (136.4-183.7) | 379 | 137.3 (117.1-161.1) | 156 | 59.3 (46.5-75.6) |
| 30-34 | 262,233 | 815 | 281.1 (253.2-312) | 693 | 239.3 (213.6-268) | 658 | 225.6 (200.8-253.5) | 580 | 204.7 (181.1-231.4) | 275 | 96.6 (80.8-115.5) |
| 35-39 | 305,626 | 1,115 | 328.6 (300.5-359.3) | 933 | 272.8 (247.3-300.9) | 888 | 258.2 (233.4-285.6) | 772 | 227.7 (204.5-253.5) | 376 | 110.5 (94.8-129) |
| 40-49 | 548,536 | 2,163 | 357 (338.2-376.8) | 1,782 | 290 (273.1-308) | 1,692 | 274.4 (258-291.9) | 1,519 | 249.2 (233.6-265.9) | 706 | 116.3 (105.8-127.9) |
| ***Norway*** | | | | | | | | | | | |
| 15-24 | 493,430 | 516 | 68.2 (59.8-77.9) | 487 | 65.1 (56.9-74.5) | 436 | 57.2 (49.6-66.1) | 498 | 65.7 (57.4-75.2) | 481 | 64.3 (56.2-73.7) |
| 25-29 | 366,926 | 1,014 | 235.1 (214-258.4) | 969 | 223.1 (202.5-245.7) | 899 | 206.2 (186.4-228) | 979 | 226.7 (206-249.6) | 959 | 220.6 (200.1-243.1) |
| 30-34 | 369,236 | 1,508 | 357.2 (330.8-385.8) | 1445 | 342.8 (317-370.8) | 1,343 | 319.2 (294.3-346.2) | 1468 | 348.2 (322.2-376.4) | 1432 | 340.5 (314.7-368.4) |
| 35-39 | 354,725 | 1,842 | 470.8 (439.9-504) | 1,788 | 455.9 (425.5-488.6) | 1,675 | 425.8 (396.4-457.4) | 1806 | 460.6 (430-493.4) | 1,778 | 452.4 (422.1-485) |
| 40-49 | 544,027 | 3,715 | 624.8 (599.8-651) | 3,617 | 609.6 (584.8-635.4) | 3,411 | 574.2 (550.2-599.2) | 3653 | 614 (589.2-640) | 3,597 | 605.5 (580.8-631.2) |
| ***United-Kingdom (Wales)*** | | | | | | | | | | | |
| 15-24 | 237,210 | 107 | 29.7 (22.1-39.9) | 73 | 19.4 (13.5-28) | 56 | 12.7 (8.1-19.9) | 76 | 21.4 (15.1-30.3) | 71 | 18.7 (12.9-27.1) |
| 25-29 | 157,805 | 204 | 112 (90.9-137.9) | 157 | 81 (63.4-103.4) | 118 | 62 (46.9-82) | 165 | 85.6 (67.4-108.6) | 151 | 78.5 (61.2-100.6) |
| 30-34 | 144,119 | 287 | 164.3 (136.5-197.8) | 214 | 121.2 (97.7-150.4) | 159 | 90.7 (70.7-116.4) | 232 | 132.3 (107.6-162.7) | 208 | 119.7 (96.3-148.7) |
| 35-39 | 131,950 | 374 | 261 (224.6-303.3) | 294 | 201.9 (170.2-239.5) | 242 | 152.8 (125.6-185.9) | 308 | 213.8 (181.1-252.4) | 287 | 197.8 (166.5-235.1) |
| 40-49 | 202,795 | 902 | 411.3 (378.3-447.3) | 691 | 310.1 (281.6-341.5) | 548 | 241.7 (216.6-269.6) | 733 | 334.2 (304.6-366.8) | 673 | 305.8 (277.5-337) |

****Supplementary t****able 9. Number of MS cases and period prevalence per 100,000 women in the 2015-2019^1^ period stratified by age group in data sources with pregnant women

|  |  | **MS1** | | **MS2** | | **MS3** | | **MS4** | | **MS5** | |
| --- | --- | --- | --- | --- | --- | --- | --- | --- | --- | --- | --- |
| Age group | Population | Cases | Prevalence | Cases | Prevalence | Cases | Prevalence | Cases | Prevalence | Cases | Prevalence |
| ***Finland*** | | | | | | | | | | | |
| 15-24 | 45,179 | 47 | 104 (78.2-138.3) | 44 | 97.4 (72.6-130.7) | 37 | 81.9 (59.4-112.9) | 40 | 88.5 (65-120.5) | 40 | 88.5 (65-120.5) |
| 25-29 | 75,093 | 133 | 177.1 (149.5-209.8) | 119 | 158.5 (132.5-189.6) | 111 | 147.8 (122.8-178) | 113 | 150.5 (125.2-180.9) | 112 | 149.1 (124-179.4) |
| 30-34 | 82,653 | 229 | 277.1 (243.5-315.3) | 214 | 258.9 (226.5-295.9) | 199 | 240.8 (209.6-276.6) | 202 | 244.4 (213-280.4) | 201 | 243.2 (211.8-279.2) |
| 35-39 | 45,846 | 134 | 292.3 (246.9-346) | 126 | 274.8 (230.9-327.1) | 114 | 248.7 (207-298.6) | 120 | 261.7 (219-312.9) | 120 | 261.7 (219-312.9) |
| 40-49 | 11,866 | 34 | 286.5 (205.1-400.1) | 30 | 252.8 (177.2-360.7) | 27 | 227.5 (156.4-330.9) | 26 | 219.1 (149.6-320.9) | 26 | 219.1 (149.6-320.9) |
| ***France (Haute-Garonne)*** | | | | | | | | | | | |
| 15-24 | 6,961 | <5 | NC | <5 | NC | <5 | NC | <5 | NC | <5 | NC |
| 25-29 | 18,155 | 15 | 82.6 (50.1-136.3) | 11 | 60.6 (33.8-108.5) | 5 | 27.5 (11.8-64.5) | 10 | 55.1 (29.9-101.4) | <5 | NC |
| 30-34 | 20,222 | 23 | 113.7 (75.8-170.6) | 16 | 79.1 (48.7-128.5) | 13 | 64.3 (37.6-110) | 13 | 64.3 (37.6-110) | <5 | NC |
| 35-39 | 9,648 | 10 | 103.6 (56.3-190.7) | 7 | 72.6 (35.2-149.7) | 5 | 51.8 (22.1-121.3) | 5 | 51.8 (22.1-121.3) | <5 | NC |
| 40-49 | 1,995 | 6 | 300.8 (137.9-654.6) | 5 | 250.6 (107.1-585.4) | <5 | NC | <5 | NC | <5 | NC |
| ***Spain (Valencian Region)*** | | | | | | | | | | | |
| 15-24 | 19,474 | <5 | NC | <5 | NC | <5 | NC | <5 | NC | <5 | NC |
| 25-29 | 40,495 | 30 | 74.1 (51.9-105.7) | 20 | 49.4 (32-76.3) | 11 | 27.2 (15.2-48.6) | 25 | 61.7 (41.8-91.1) | 6 | 14.8 (6.8-32.3) |
| 30-34 | 72,739 | 90 | 123.7 (100.7-152) | 47 | 64.6 (48.6-85.9) | 42 | 57.7 (42.7-78) | 78 | 107.2 (85.9-133.8) | 20 | 27.5 (17.8-42.5) |
| 35-39 | 59886 | 83 | 138.6 (111.8-171.8) | 48 | 80.2 (60.5-106.2) | 33 | 55.1 (39.2-77.4) | 74 | 123.6 (98.5-155.1) | 15 | 25 (15.2-41.3) |
| 40-49 | 16352 | 24 | 146.8 (98.7-218.3) | 12 | 73.4 (42-128.2) | 8 | 48.9 (24.8-96.5) | 20 | 122.3 (79.2-188.9) | 5 | 30.6 (13.1-71.6) |

## ^1^ 2015-2018 for Finish data source; NC= Non-calculable

****Supplementary table 10****. Published MS prevalence in women in the areas covered in our study

| Country | Region | Article | Prevalence in women | Prevalence per 100,000 women | Period | Population/setting | Identification of MS cases |
| --- | --- | --- | --- | --- | --- | --- | --- |
| Italy | Ferrara | Granieri et al., 2018 | Yes | 261 | 2016 | Population from the province of Ferrara, cases identified from administrative health data and medical records | Manual review of patient records and clinical assessments based on Poser’s diagnostic criteria and EDSS |
| Norway | Nordland | Benjaminsen et al., 2014 | Yes | 250 | 2010 | Patient living in Nordland County treated at hospital | MS diagnosis based on Poser or McDonald criteria for definite or probable MS |
| Norway | Nationwide | Berg-Hansen et al., 2014 | Yes | 280 | 2012 | Patients registered in a national registry covering secondary care | MS diagnosis based on Poser or McDonald criteria, or at least 2 MS records for cases registered in the NPR |
| Norway | Hordaland | Grytten et al., 2016 | Yes | 271 | 2013 | Patients from previous studies (until 2003) and from hospital patient records | MS diagnosis based on data from neurological centres using Poser and McDonald criteria |
| Norway | Buskerud | Simonsen et al., 2017 | Yes | 293 | 2013 | Patients hospitalized living in Buskerud | MS diagnosis based on Poser criteria for definite or probable MS before 2001, or McDonald criteria for MS after 2002 |
| Norway | Møre and Romsdal | Willumsen et al., 2020 | Yes | 473 | 2018 | Patients with MS in Møre and Romsdal, identified using local, regional, and national sources | MS diagnosis made by a clinical neurologist; consensus reached by the first and last author in unclear cases |
| Norway | Telemark | Flemmen et al., 2020 | Yes | 347 | 2019 | Patients residing in Telemark County, identified from hospital patient records | MS diagnosis based on ICD-10 for MS and diagnostic criteria for definite or probable MS |
| United-Kingdom | Wales | Mackenzie et al., 2014 | Yes | 243 | 2010 | Population included in the GPRD | MS diagnosed based on Read codes (GPRD) and ICD10 codes (HES) |
| United-Kingdom | Wales | Nicholas R, et al., 2024 | No | 222 (in men and women) | 2020 | Population registered in the SAIL databank | Algorithm requiring an MS diagnosis code 6 months after the first entry, or 3 diagnosis codes |
| Finland | Nationwide | Laakso et al., 2019 | Yes | >260 | 2018 | Patients from national MS registry | MS diagnoses identified through manual data entry or integration from hospital data, verified by chart review |
| France | Haute-Garonne | Foulon et al., 2017 | Yes | 196 | 2012 | Population from the French  National Health Insurance database | Algorithm requiring on one event among LTD Status for MS status, hospitalization, or MS-specific drug reimbursement. |
| Spain | Valencian Region | Cayuela et al., 2024 | Yes | 153 | 2021 | Patients from the Primary Care Clinical Database of the National Health System | MS cases identified using ICPC-2 code for MS |
| United-States | Michigan | MacDonald et al., 2018 | Pregnant women | 130 | 2011-2015 | Pregnant women from the Truven Health cohort | ICD-9-CM code recorded on at least 2 unique days from 90 days before LMP to the delivery date |

MS: Multiple Sclerosis, EDSS: Expanded Disability Status Scale, NPR: Norwegian Patient Registry, GPRD: General Practice Research Database, HES: Hospital Episode Statistics LTD: Long-Term Disease; LMP: Last Menstrual Period
